# Further Clinical Validation of the Walking Impairment Questionnaire for Classification of Walking Performance in Patients with Peripheral Artery Disease

**DOI:** 10.1155/2012/190641

**Published:** 2012-08-02

**Authors:** S. P. Sagar, P. M. Brown, D. T. Zelt, W. L. Pickett, J. E. Tranmer

**Affiliations:** ^1^Department of Community Health and Epidemiology, Queen's University, Kingston, ON, Canada K7L 3N6; ^2^Division of Vascular Surgery, Department of Surgery, Kingston General Hospital, Kingston, ON, Canada K7L 2V7; ^3^Department of Emergency Medicine, Kingston General Hospital, Kingston, ON, Canada K7L 2V7; ^4^School of Nursing, Queen's University, Kingston, ON, Canada K7L 3N6

## Abstract

The purpose of this study was to further validate the *Walking Impairment Questionnaire* (WIQ) as a self-report tool to aid in the clinical identification of walking ability of patients with peripheral artery disease (PAD). 132 patients with PAD and an ankle brachial index (ABI) ≤0.90 were enrolled; 123 provided complete data for the WIQ and standardized graded treadmill test. The WIQ scores were consistent with reported scores in other studies. The absolute claudication distance (ACD) ranged from 42.3 to 1589.2 meters; the peak walking time (PWT) ranged from 68 to 1800 seconds. Adjusted WIQ scores were positively and moderately associated with the log transformed ACD and PWT (*r* > .53, *P* < .001). Based on the area under the curve analysis, an overall WIQ score of 42.5 or less identified low performers (sensitivity 0.90, specificity 0.73); the combined subscale score of distance and stair of 75.5 or more identified high performers (sensitivity 0.41, specificity 0.90). We conclude that WIQ cut-offs appropriately classify walking performance in PAD patients, making this a potentially useful clinical tool. Consideration needs to be given to incorporating a standardized WIQ version into practice guidelines and the use of innovative strategies to facilitate clinical uptake.

## 1. Introduction

Peripheral artery disease (PAD) is a prevalent chronic condition that increases with age, affecting 20% of persons over the age of 75 years, and is associated with exceptionally high risks for cardiac and cerebrovascular events [1, 2]. Intermittent claudication, defined as the onset of pain in the leg or gluteal muscles with exertion which resolves with rest, is a sentinel symptom of PAD and, in most cases, indicative of disease severity. The effects of claudication on walking performance, that is the ability to walk without pain, vary within patients who have similar clinical profiles [3–6], suggesting that there are other factors that influence walking performance. The primary goal of conservative clinical management of PAD is to minimize disease progression and optimize performance; thus, the ability to easily evaluate the effects of treatment, including lifestyle modification, on walking performance is clinically important. Walking performance has been measured via patient-report questionnaire tools [7–12] or standardized treadmill tests [13, 14]. However, many clinicians may not use these measures and rely solely on patient's subjective responses to their questioning. Thus it is difficult to quantify, monitor and accurately assess actual or changes in performance across the continuum of this chronic condition.

Walking performance in patients with PAD has been assessed with continuous and graded treadmill tests; the graded test is a more reliable measure of performance in patients with PAD [15]. Patients participate in a standardized protocol and walk on the treadmill at increasing elevations until they experience pain [14]. A number of measures are obtained, including the absolute claudication distance (ACD) defined as the maximal distance the patient can reach before they can no longer walk on the treadmill because of claudication pain or peak walking time (PWT) defined as the greatest time of exercise achieved [16]. While these tests provide accurate assessment of the impact of claudication on walking performance and are considered the gold standard of assessment, they are not feasible to conduct in most clinical settings. 

The *Walking Impairment Questionnaire* is a common patient-reported measure, first developed by Regenstiener et al. [7], designed to assess walking ability in patients with PAD [7–10]. The WIQ has been used to describe walking performance [9] and to assess the efficacy of clinical interventions [10]. The tool has been translated and employed in different countries [17–19] and in different conditions [20]. The tool can be either interview administered or completed by the patient [21], with no significant impact on responses. Concerns have been raised about the complexity of the wording of some of the items on the tool (i.e., patients rate a lower difficulty for higher intensity tasks) and the need to correct responses [22]. While these studies have provided important normative data and descriptions and have suggested that this tool could be used to clinically monitor walking performance, the WIQ remains predominantly a research tool. 

Therefore, the primary aim of this study was to further validate the WIQ as a clinical tool for patients with peripheral artery disease. Specifically, we hoped to identify valid cut-off points for identifying patients with low and high walking ability, as indicated by the WIQ. Categorization of high and low performers, in combination with knowledge of a patient's clinical condition could allow clinicians to more effectively prescribe treatment strategies for patients' symptoms, monitor progress and make changes to patient management as needed. 

## 2. Methods

### 2.1. Participant Identification and Selection

The research protocol was reviewed and approved by the Queen's University Health Sciences Research Ethics Board. All consecutive PAD patients seen in the vascular clinic at Kingston General Hospital between May 2010 and May 2011 who met the inclusion criteria were identified by two attending vascular surgeons. The identified patients were telephoned, consented and invited to return to the hospital for a study visit. The study design was cross-sectional.

### 2.2. Inclusion and Exclusion Criteria

Patients were included if they had a resting ankle-brachial index (ABI) of ≤0.90. Participants were excluded if they had (a) severe ischemia requiring intervention, (b) comorbid conditions that limited walking (angina, congestive heart failure, chronic obstructive pulmonary disease or severe arthritis), (c) wheel chair, cane or walker requirement, (d) non-compressible arteries, and/or (e) severe cognitive impairment. The exclusion criteria were selected to ensure that participants were able to walk safely on a treadmill, and to ensure that claudication due to PAD was the limiting factor for walking performance. 

#### 2.2.1. Treadmill Test

The treadmill test was similar to protocols followed in previous PAD studies [23, 24] and consisted of a progressive, graded treadmill protocol (constant speed at 3.2 km/hr after initial increase, 0% grade initially with 2% increases in grade every two minutes after the initial speed increase to a maximum of 10%) conducted until maximal claudication pain was reached or to a maximum duration of 30 minutes (about 1.6 km). The peak walking time (PWT) and distance of absolute claudication (ACD) were identified as the time and distance to maximal pain, classified as 8/10 on the BORG perceived exertion scale [25]. Participants familiarized themselves with the treadmill by starting at an initial speed of 1.7 km/hr. This was increased by 1.6 km every 10 seconds for the first 90 seconds until the maximum speed of 3.2 km/hr was reached. The test start time was the beginning of the treadmill testing session (i.e., included familiarization and graded protocol). Participants were excluded from the analysis if they failed to complete the treadmill test for reasons other than claudication, such as shortness of breath.

#### 2.2.2. Walking Impairment Questionnaire

The *Walking Impairment Questionnaire* utilized in this study contained 14 items that contributed to 3 subscales (distance, speed and stair) and an overall score. A copy of the questionnaire is included in the Appendix. It should be noted that this version of the WIQ differs from the original version first validated by Regensteiner et al. [7]; the distance scale includes one more level; the speed questions are the same and there is an added stair subscale. The scoring algorithms are similar. Participants answer each item on a Likert scale from 0 for “unable to do” to 4 for “no difficulty”. Each response is weighted based on the difficulty of the task (e.g., the weight for “walk slowly” is 1.5 whereas for the weight for “run or jog” is 5). Subscale scores are determined by dividing the weighted answers by the maximum possible weighted score and multiplying by 100. Each score ranges from 0–100 with lower scores indicating lower performance. The overall score is the average of all 3 subscores and combined scores are the average of 2 distinct subscales (e.g., distance and speed, distance and stairs, speed and stairs). Items coded as “Didn't do for other reasons” or missing are removed from the denominator of the weighted score to calculate a percent score based on the items that remained (i.e., limitation, if any, was due only to intermittent claudication). In example, if a participant responded with much difficulty for walking 1500 feet (5 blocks), some difficulty for walking 900 feet (3 blocks) and no difficulty for the remaining distance questions the distance score would be: [4(20) + 4(50) + 4(150) + 4(300) + 4(600) + 3(900) + 1(1500)]/14080∗100 = (8680/14080)∗100 = 61.65. If more than half of the items in a subscale are coded as such the subscore is coded as missing [4]. 

In this study, participants completed the questionnaire after the treadmill test session, after a rest period. Participants were instructed to select the answer they felt was most appropriate for them. The administrator provided no additional guidance to the participant. When reviewing the questionnaire responses we noted that some participants seemed to misunderstand the “around the home” question and based their answer on the presence of stairs in the house rather than the ability to walk on level ground. We corrected for this by recoding these cases to the “50 feet” category if the participant's answer for “around the home” was lower. 

#### 2.2.3. Other Variables

The ABI was obtained from previous testing completed at the Vascular Testing Centre, within 6 months of testing. Brachial pressure and 2 pedal pressures (right and left) are determined. The lowest ABI was used as the participant's ABI. Weight and height were measured using a medical scale to determine the participant's body-mass index (weight over height squared). Waist circumference was measured at the top of the iliac crest using anthropometric tape. Diabetic status (yes or no), smoking status (current, former or never) were self reported. Age was determined based on the participant's self-reported birth date and year of testing date. Number of pack years was determined based on the number of cigarettes smoked daily divided by 20 (standard pack size) multiplied by the estimated duration of smoking in years. 

#### 2.2.4. Statistical Analysis

The sample was initially profiled using conventional descriptive statistics. Comparison of the ABI between those who participated and those contacted and who did not participate was determined using two-sample independent *t*-tests. Raw and log transformed estimates were obtained for ACD and PWT. Scores for each subscale, combined subscales and overall scale of the *Walking Impairment Questionnaire* were determined. Participants' walking performance was classified as low, medium and high based on the distribution of the ACD scores by tertiles. Receiver operating characteristic (ROC) curves were generated for identifying high and low walking ability for each subscale score, combined and overall scale scores. These curves were obtained by plotting the sensitivity against 1-specificity using 0.5 score increments of the scale scores. The area under the curve was calculated using the trapezoidal method [26]. The cut-off values for the questionnaire were identified for varying levels of sensitivity and specificity (at least 0.80 and at least 0.90). Positive and negative predictive values for the cut-offs of the score with the highest area under the ROC curve for 0.90 sensitivity for low performers and 0.90 specificity for high performers were calculated. 

## 3. Results

**Figure 1:**
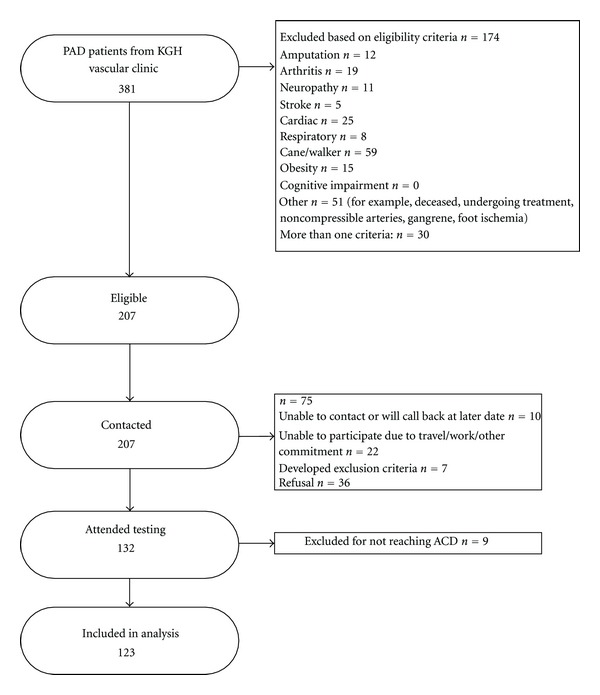
Participant flow.

174 of the 381 PAD patients screened were deemed ineligible based on the exclusion criteria. Of the 207 eligible patients 132 (63.8%) patients consented and participated in testing. 8 participants stopped the test prior to the onset of claudication (e.g., due to shortness of breath) and one additional patient stopped prior to maximum claudication. 123 patients were, therefore, included in the analysis (Figure 1). 

### 3.1. Participant Characteristics

The characteristics of the PAD patients who participated in the study (*n* = 123) are described in Table 1. There was no significant difference in ABI between those who participated and those contacted who did not participate (Mean ABI, 0.58 and 0.60 resp.). In this study, 11 participants self-reported no claudication; 9 of these 11 experienced claudication with treadmill testing. 

### 3.2. Walking Impairment Questionnaire Scores

**Figure 2:**
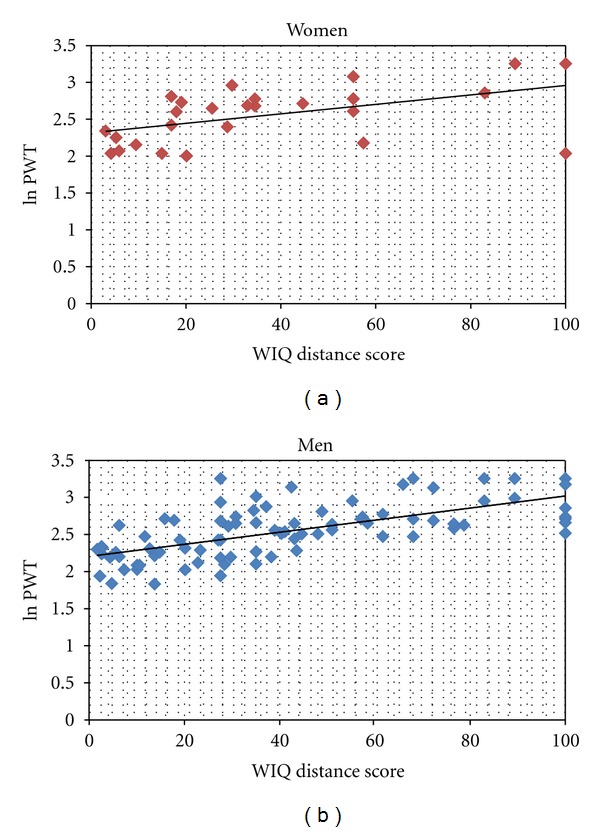
Distribution of WIQ distance scores by PWT within men and women.

The subscale and overall WIQ scores, categorized according to the ACD tertiles are shown in Table 2. Due to missing data, sample sizes vary for each of the subscores. The scores ranged from a 0 to 100. The scores increased consistently across the three performance groups, as classified by the ACD obtained via the graded treadmill test. Despite a large standard deviation in scores within each performance group, all comparisons achieved a high level of statistical significance. In a bivariate analysis (data not shown) men reported higher WIQ speed scores in comparison to women (*P* = 0.02); there were no other sex difference in other subscale scores, ACD and PWT. As well, there were no significant age differences in the WIQ scores when age was dichotomized at 60 and above. The associations between the ACD, PWT and WIQ scores, when controlling for age, sex, and ABI were all moderate to strong (partial correlation coefficient, *r* > 0.5, *P* < .001). (See Table 3) Figure 2 plots the linear trend model between the WIQ distance scores and PWT (log transformed) within each sex category.

### 3.3. Identifying Cut-Offs for Low Walking Performance

The area under the curve of the receiver operating characteristics (ROC) curve provides information about the ability of a test to identify true positives and true negatives. The closer the area under the curve is to 1, the better the test is at distinguishing between patient groups. In all analyses we used the unadjusted ACD score to identify non-performers and performers as our focus was on actual walking distance; as well the ACD and PWT scores (transformed and non-transformed) were highly correlated (*r* ≥ 0.92). The area under the curve values for the ROC ranged from 0.80 to 0.89 with the value for the overall WIQ score providing the highest value (Table 4). Based on this analysis, a WIQ overall score of less than or equal to 39.0 permitted identification of a low performer with a sensitivity of at least 0.80 while maximizing specificity (0.75). A WIQ overall score of 42.5 increased the sensitivity to at least 0.90 but decreased the specificity to 0.73. Similar cut-off values are shown for 0.8 specificity and 0.9 specificity in Table 4.

### 3.4. Identifying Cut-Offs for High Walking Performance

The area under the curve values for the ROC ranged between 0.73 and 0.81, with the value for the combined distance and stair climbing ability being the highest (Table 4). A combined distance and stair climbing ability score of 58.0 permitted identification of a high performance with a specificity of at least 0.80. Choosing a cut-off of 75.5 increased the specificity to at least 0.90 but decreased the sensitivity to 0.41. Similar cut-off values are shown for 0.8 sensitivity and 0.9 sensitivity in Table 5. The area under the curve values for identifying high walking performance were lower than those for identifying low walking performance. 

### 3.5. Predictive Values

Negative predictive values were higher for low performers (0.94) in comparison to high performers (0.75). In both cases the positive predictive value was lower (0.62 for identifying low performers, 0.70 for identifying high performers).

## 4. Discussion

The ability to objectively measure walking performance in patients with PAD with a range of claudication symptoms is relevant to conservative management [27]. While recent guidelines acknowledge the ability of treadmill tests to objectively quantify performance, it is not recommended as a routine measure for practice [28]; thus the need to consider less invasive yet reliable tools. The *Walking Impairment Questionnaire* (WIQ) is the most commonly reported self-report tool that has been used to evaluate patient's walking ability. Although it is typically used as a research tool, it is also has potential to be applied to routine clinical management of patients with PAD. We, therefore, built upon previous research and determined cut-off values for the WIQ for the potential classification of low and high walking performance in a diverse PAD patient population; information that could easily be used by clinicians to make more informed decisions concerning a patient's treatment plan.

The WIQ scores reported in this study (39.5 for distance, 47.6 for speed and 58.0 for stair climbing) are similar to those of previous studies which ranged from 38 to 55 for distance, 37 to 52 for speed and 48 to 68 for stair climbing [8–10, 29]. In our sample, the mean ACD was 418 meters, similar to other studies with similar populations [23, 24]. Thus both of our standard measures of walking performance are consistent with the most current reports.

Previous studies have reported moderate to strong correlations between scores on the WIQ and walking performance as measured by treadmill tests in patients with intermittent claudication [9, 30, 31]. Regensteiner et al. reported that the WIQ distance and speed scores correlated moderately and significantly with the peak treadmill walking time (PWT) (*r* = 0.68, *P* < 0.05, *n* = 26) [7]; Myers et al. reported significant and moderate correlations between the WIQ distance and speed scores and the ACD (Spearman's rank correlations 0.41 and 0.39, resp., *P* < 0.05, *n* = 48) [9]; and Verspaget et al., using a Dutch version of the questionnaire, reported similar correlations: distance, speed and stair climbing scores as well as the overall score were moderately correlated with the ACD (0.45, 0.43, 0.37, 0.52, resp., all *P* < 0.01, *n* = 130) [29]. Our findings support these previous associations and the linear association between patient-reported scores and PWT and ACD (data not shown). 

### 4.1. Identification of High and Low Performers

While the correlation values suggest that moderate to strong associations exist between self-report assessment and actual walking ability, these do not provide clinicians with cut points or indicators of performance. Based on the area under the curve of the ROC analysis, we were able to determine that the overall WIQ score was the most appropriate score for identifying low performers while the combined distance and stair score was the most appropriate for identifying high performers. However, the 95% confidence intervals of the area under the curve of the ROC for all scores or combination of scores overlap. Thus there may not be a significant difference between the accuracy of a particular score or combination of scores in classifying performance. Since no score appears to be significantly more accurate than another, consideration could be given to consistently using the overall WIQ score. 

The accurate identification of low performers is important as these patients' symptoms are impacting walking performance more. It is therefore important to have low false negatives. This translates into a test for identifying low performers with high sensitivity. In our study, to obtain a sensitivity of at least 0.80 or 0.90 the cut-off values for the overall score were 39.0 (specificity = 0.77) and 42.5 (specificity = 0.73), respectively. Thus, with a cut off score of 42.5 or less we could identify 90% of low performers (i.e., participants who were only able to walk, on average,.96.6 meteres). With this score we would also identify 30% of the participants who were actually performing well but scored lower on the WIQ. From a clinical perspective, this would be reassuring as we would accurately assess patients as low performers most of the time and plan care accordingly. Misclassification of patients who are actually performing would have minimal clinical impact. 

High performers may not require further invasive or different interventions as their current conservative management and lifestyle (i.e., exercise) is adequate. Therefore, it is important to have a test with high specificity for identifying high performers with low false positives to ensure that low performers are identified and receive the intervention that they need. To obtain a specificity of at least 0.80 or 0.90, the cut-off values for the combined distance and stair score were 58.0 (sensitivity = 0.62) and 75.5 (sensitivity = 0.41), respectively. Less than 10% of low performers would have a combined distance and stair scores of 75.5 or more; however, 59% of high performers would be identified as being low performers with that same cut-off. Again, from a clinical perspective these cut-offs would be reassuring.

The cut-off value for identifying low performers had both high sensitivity and specificity (0.90 and 0.73). It also had a very high negative predictive value (0.94) but a lower positive predictive value (0.62) indicating that this score was very good at identifying low performers in this population but may result in the overtreatment of patients who are misclassified as low. The cut-off value for identifying high performers had high specificity (0.90) but low sensitivity (0.41). In this population it had high positive predictive value and high negative predictive value (0.70 and 0.75, resp.) indicating that, despite a low specificity, the cut-off may be effective at differentiating between high performers and non-high performers. The population used in this study appears to be a clinically diverse patient sample reflective of the typical PAD population; therefore, the positive and negative predictive values identified may be generalizable to the greater PAD population.

### 4.2. Strengths and Limitations

The specific strengths of this study were as follows: validation testing in a large, clinically diverse patient sample reflective of the typical PAD population; comparison of the WIQ with a graded treadmill test; analysis of varied score combinations for the WIQ and detailed ROC curve analysis to determine clinically useful cut-off values. There were limitations, however. One limitation of the study is the questionnaire design itself. Approximately twenty participants perceived they had a higher level of difficulty walking around their home than walking 50 feet. Participants commented that in-home walking ability included stair climbing and this was more difficult than walking on level ground as the question states. Adjusting for this was, therefore, done as described in the methods. This problem, highlighted in previous research [22] needs to addressed through item or question modification. Further testing is warranted. The timing of questionnaire administration after the treadmill test may have influenced the participant's responses. Participants who were pleased with their treadmill test results may have overinflated their ability; participants who were not pleased may have scored lower. Regardless, self reports of patient activity are reliable estimates of activity [32, 33]. Repeat administration of the WIQ, perhaps during clinical follow-up, would allow for comparison of reported scores. 

The population studied, while a diverse population of PAD patients (from severe impairment to no claudication), was limited to individuals able to safely participate in a treadmill test and whose walking was limited by claudication and not other factors. The generalizability of the results is restricted to this group. This was also a strength of the study as the findings are generalizable to a group who could potentially participate in exercise interventions designed to alleviate symptoms and promote performance. A large number of patients either refused or were unable to participate; however, they were not clinically different than those who did participate. The high number of patients unwilling to participate in a treadmill test or unable to attend a test date does highlight the importance of a having a non-invasive tool such as the WIQ with established cut-off points for use in future studies and/or practice.

## 5. Conclusions 

Our findings further support that the WIQ could be used to classify the walking performance of patients with PAD in a clinical setting, with an acceptable level of sensitivity and specificity. In a diverse population of patients able to safely participate in a treadmill test, an overall WIQ score of 43 or less identified low performers. An overall WIQ score of 70 or higher or a combined distance and stair score of 76 or higher similarly identified high performers. Individuals in the middle range of these scores could also be classified as “moderate performers”, with an opportunity to improve. This ability to classify walking performance, a sentinel indicator of disease impact, when combined with other patient characteristics could inform clinical decisions and guide patient management. Given the documented evidence that daily activity and exercise enhances walking performance [24, 34–36] clinicians need tools to assess and monitor progress and/or decline. Reasons behind the poor uptake of validated self-report measurement tools in the clinical setting are complex, but likely related to factors such as the: (a) validity and usefulness of the results, (b), ease of application and (c) integration of assessment tool into current clinical flow. Integration of validated self-report measures, through a variety of means (i.e., electronic kiosks) are becoming an important component of clinical symptom management and practice in other conditions and settings [37, 38]. Similar strategies could be employed in specialty vascular clinics or the primary care setting. The WIQ has been in existence since 1990. The poor uptake of a valid measure of an important symptom for patients with PAD is concerning. Further research may need to explore the development and validation of revised and shorter versions of the WIQ in similar patient populations. However, a standardized version of the tool for adoption into clinical practice guidelines would be helpful for clinicians, and likely facilitate uptake. As well, a cohort or natural history study of patients with the recommended cut-offs should be conducted to assess the prognostic potential and clinical utility of the suggested WIQ cut-offscores.

## Figures and Tables

**Table 1:** Baseline characteristics of participants.

Characteristics	
*N*	123
Age (years)—mean (SD)	66.5 (9.4)
Male sex %	70.7
ABI—mean (SD)	0.61 (0.17)
BMI (kg/m^2^)—mean (SD)	28.0 (5.6)
Waist circumference (cm)—mean (SD)	98.5 (12.9)
Current smoker %	31.4
Former smoker %	65.3
Never smoked %	3.3
Pack years—mean (SD)	23.8 (14.3)
Diabetes %	22.8

SD: standard deviation, ABI: ankle brachial index, BMI: body mass index.

**Table 2:** WIQ scores categorized by treadmill walking performance as measured by the ACD.

	*n*	Low		Medium		High		All		*P*
		Mean	SD	Mean	SD	Mean	SD	Mean	SD	
Absolute claudication distance (meters)	123	103.0	32.0	296.8	79.2	849.9	423.0	416.5	402.5	<0.0001^∗^
Peak walking time (seconds)	123	137.6	36.0	354.4	88.6	973.0	473.1	488.4	450.2	<0.0001^∗^
WIQ-distance	119	18.3	19.4	38.4	25.3	59.7	27.8	39.5	30.2	<0.0001^∗^
WIQ-speed	119	27.9	20.6	49.9	25.8	61.3	24.9	47.6	28.0	<0.0001^†^
WIQ-stair	109	37.5	21.3	61.4	26.9	73.8	23.0	58.0	27.8	<0.0001^†^
WIQ-distance and speed	115	23.2	19.2	44.6	22.0	60.1	23.7	39.5	30.2	<0.0001^∗^
WIQ-overall	102	26.9	13.8	52.0	21.2	65.0	20.6	47.6	28.0	<0.0001^∗^

SD: standard deviation.

Low (<159.89 meters), medium (159.89–415.89 meters), high (>415.89 meters) categories are based on thirds of the population divided by tertiles of ACD.

*P  *value: ANOVA test.

^∗^Significant difference between all groups using Bonferroni method.

^†^Significant difference between all groups except medium and high using Bonferroni method.

Note: sample sizes vary for each subscore based on the number who had fewer than half missing values for that subscores. If any of the subscores were missing the overall score was coded as missing.

**Table 3:** Partial correlation coefficients (*r*) of the scores of the *Walking Impairment Questionnaire* relative to peak walking time (ln PWT).

WIQ score	*r*
Distance	0.63
Speed	0.69
Stair	0.53
Distance and speed	0.69
Distance and stair	0.65
Speed and stair	0.66
Overall	0.69

All correlations were adjusted for age, sex, and ABI.

All correlations were significant (*P* < .001).

**Table 4:** Cut-offs for the WIQ subscores and combined scores for various sensitivity and specificities as well as the area under the curve of the ROC for identifying those in the *low *walking performance group.

	0.8 sensitivity		0.9 sensitivity		0.8 specificity		0.9 specificity		Area under the curve of the ROC (95% CI)
	Cut-off	Specificity	Cut-off	Specificity	Cut-off	Sensitivity	Cut-off	Sensitivity	
Distance	28.5	0.73	38.5	0.57	25.0	0.73	15.5	0.62	0.83 (0.75–0.91)
Speed	39.5	0.66	58.0	0.39	35.5	0.66	24.5	0.50	0.81(0.72–0.90)
Stair	54.5	0.65	67.0	0.47	41.5	0.47	29.0	0.33	0.81 (0.73–0.89)
Distance and speed	33.5	0.73	44.5	0.57	30.5	0.74	27.0	0.69	0.85 (0.77–0.93)
Distance and stair	39.0	0.76	47.0	0.69	36.0	0.76	28.5	0.48	0.86 (0.79–0.93)
Speed and stair	44.0	0.79	50.0	0.69	42.5	0.74	32.5	0.53	0.88 (0.81–0.94)
**Overall**	**39.0**	**0.77**	**42.5**	**0.73**	**35.0**	**0.72**	**32.5**	**0.66**	**0.89 (0.82**–**0.95)**

ROC: receiver operating characteristics.

CI: confidence interval.

The bold score has the highest area under the curve of the ROC.

**Table 5:** Cut-offs for the WIQ subscores and combined scores for various sensitivities and specificities as well as the area under the curve of the ROC for identifying those in the *high* walking ability group.

	0.8 sensitivity		0.9 sensitivity		0.8 specificity		0.9 specificity		Area under the curve of the ROC (95% CI)
	Cut-off	Specificity	Cut-off	Specificity	Cut-off	Sensitivity	Cut-off	Sensitivity	
Distance	30.5	0.63	19.0	0.42	44.0	0.66	62.0	0.45	0.80 (0.72–0.89)
Speed	39.0	0.55	31.5	0.44	57.5	0.49	83.0	0.26	0.74 (0.65–0.83)
Stair	54.0	0.51	41.5	0.36	67.0	0.60	87.5	0.23	0.76 (0.66–0.85)
Distance and speed	36.0	0.63	28.0	0.44	53.0	0.50	64.0	0.47	0.78 (0.70–0.87)
**Distance and ** **stair**	**47.0**	**0.68**	**38.0**	**0.56**	**58.0**	**0.62**	**75.5**	**0.41**	**0.81 (0.73**–**0.90)**
Speed and stair	48.0	0.61	36.0	0.38	64.0	0.57	75.0	0.46	0.78 (0.69–0.88)
Overall	44.0	0.67	34.0	0.47	61.0	0.53	69.5	0.47	0.80 (0.72–0.89)

ROC: receiver operating characteristics.

CI: confidence interval.

The bold score has the highest area under the curve of the ROC.

**Table 6:** Distance subscale of the WIQ.

Please place a *√* in the box that best describes how hard it was for you to walk on level ground without stopping to rest for each of the following distances during the last week:							

During the last week, how difficult was it for you to:	No difficulty	Slight difficulty	Some difficulty	Much difficulty	Unable to do	Did not do for other reasons	Weight
a. Walk indoors, such as around your home?	□	□	□	□	□	□	20
	4	3	2	1	0		
b. Walk 50 feet?	□	□	□	□	□	□	50
	4	3	2	1	0		
c. Walk 150 feet? (1/2 block)?	□	□	□	□	□	□	150
	4	3	2	1	0		
d. Walk 300 feet? (1 block)?	□	□	□	□	□	□	300
	4	3	2	1	0		
e. Walk 600 feet? (2 blocks)?	□	□	□	□	□	□	600
	4	3	2	1	0		
f. Walk 900 feet? (3 blocks)?	□	□	□	□	□	□	900
	4	3	2	1	0		
g. Walk 1500 feet? (5 blocks)?	□	□	□	□	□	□	1500
	4	3	2	1	0		

**Table 7:** Speed subscale of the WIQ.

Please place a *√* in the box that best describes how hard it was for you to walk on level ground at each of these speeds without stopping to rest during the last week. Please note 1 block is roughly equivalent to 300 feet.							

During the last week, how difficult was it/or you to:	No difficulty	Slight difficulty	Some difficulty	Much difficulty	Unable to do	Didn't do for other reasons	weight
a. Walk 1 block slowly?	□	□	□	□	□	□	1.5
	4	3	2	1	0		
b. Walk 1 block at average speed?	□	□	□	□	□	□	2
	4	3	2	1	0		
c. Walk 1 block quickly?	□	□	□	□	□	□	3
	4	3	2	1	0		
d. Rum or jog 1 block?	□	□	□	□	□	□	5
	4	3	2	1	0		

**Table 8:** Stair subscale of the WIQ.

Please place a *√* in the box that best describes how hard it was for you to climb stairs without stopping to rest during the last week. Please note 1 flight of stairs is roughly equal to 14 steps.							

During the last week, how difficult was it for you to:	No difficulty	Slight difficulty	Some difficulty	Much difficulty	Unable to do	Did not do for other reasons	Weight
a. Climb 1 flight of stairs?	□	□	□	□	□	□	1
	4	3	2	1	0		
b. Climb 2 flights of stairs?	□	□	□	□	□	□	2
	4	3	2	1	0		
c. Climb 3 flight of stairs?	□	□	□	□	□	□	3
	4	3	2	1	0		
